# Ethyl 4,4′′-difluoro-5′-meth­oxy-1,1′:3′,1′′-terphenyl-4′-carboxyl­ate

**DOI:** 10.1107/S160053681105344X

**Published:** 2011-12-21

**Authors:** Hoong-Kun Fun, Tze Shyang Chia, S. Samshuddin, B. Narayana, B. K. Sarojini

**Affiliations:** a X-ray Crystallography Unit, School of Physics, Universiti Sains Malaysia, 11800 USM, Penang, Malaysia; b Department of Studies in Chemistry, Mangalore University, Mangalagangotri, Mangalore 574 199, India; c Department of Chemistry, P. A. College of Engineering, Nadupadavu, Mangalore 574 153, India

## Abstract

In the title compound, C_22_H_18_F_2_O_3_, the two fluoro-substituted rings form dihedral angles of 25.89 (15) and 55.00 (12)° with the central benzene ring. The eth­oxy group in the mol­ecule is disordered over two positions with a site-occupancy ratio of 0.662 (7):0.338 (7). In the crystal, mol­ecules are linked by C—H⋯O hydrogen bonds into chains along the *a* axis. The crystal packing is further stabilized by C—H⋯π and π—π inter­actions, with centroid–centroid distances of 3.8605 (15) Å.

## Related literature

For a related structure and background to terphenyls, see: Fun *et al.* (2011 ▶); Samshuddin *et al.* (2011 ▶). For the synthesis, see: Kotnis (1990 ▶). For reference bond lengths, see: Allen *et al.* (1987 ▶).
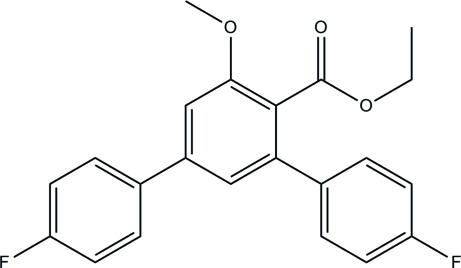


## Experimental

### 

#### Crystal data

C_22_H_18_F_2_O_3_
*M* *_r_* = 368.36
Orthorhombic, 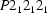

*a* = 8.5197 (11) Å
*b* = 9.5225 (12) Å
*c* = 22.871 (3) Å
*V* = 1855.5 (4) Å^3^
*Z* = 4
Mo *K*α radiation
μ = 0.10 mm^−1^
*T* = 296 K
0.43 × 0.21 × 0.15 mm

#### Data collection

Bruker APEX DUO CCD area-detector diffractometer
Absorption correction: multi-scan (*SADABS*; Bruker, 2009 ▶) *T*_min_ = 0.958, *T*_max_ = 0.986
11701 measured reflections
3036 independent reflections
2131 reflections with *I* > 2σ(*I*)
*R*_int_ = 0.026

#### Refinement

*R*[*F* ^2^ > 2σ(*F* ^2^)] = 0.057
*wR*(*F* ^2^) = 0.171
*S* = 1.10
3036 reflections
251 parameters
H-atom parameters constrained
Δρ_max_ = 0.20 e Å^−3^
Δρ_min_ = −0.23 e Å^−3^

### 

Data collection: *APEX2* (Bruker, 2009 ▶); cell refinement: *SAINT* (Bruker, 2009 ▶); data reduction: *SAINT*; program(s) used to solve structure: *SHELXTL* (Sheldrick, 2008 ▶); program(s) used to refine structure: *SHELXTL*; molecular graphics: *SHELXTL*; software used to prepare material for publication: *SHELXTL* and *PLATON* (Spek, 2009 ▶).

## Supplementary Material

Crystal structure: contains datablock(s) global, I. DOI: 10.1107/S160053681105344X/rz2682sup1.cif

Structure factors: contains datablock(s) I. DOI: 10.1107/S160053681105344X/rz2682Isup2.hkl

Supplementary material file. DOI: 10.1107/S160053681105344X/rz2682Isup3.cml

Additional supplementary materials:  crystallographic information; 3D view; checkCIF report

## Figures and Tables

**Table 1:** Hydrogen-bond geometry (Å, °) *Cg*2 and *Cg*3 are the centroids of the C7–C12 and C13–C18 rings, respectively.

*D*—H⋯*A*	*D*—H	H⋯*A*	*D*⋯*A*	*D*—H⋯*A*
C12—H12*A*⋯O2^i^	0.93	2.59	3.515 (3)	179
C5—H5*A*⋯*Cg*3^ii^	0.93	2.92	3.589 (3)	130
C20—H20*A*⋯*Cg*2^iii^	0.96	2.83	3.710 (4)	152

Symmetry codes: (i) 
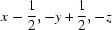
; (ii) 
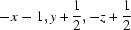
; (iii) 
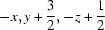
.

**Fig. 1.**
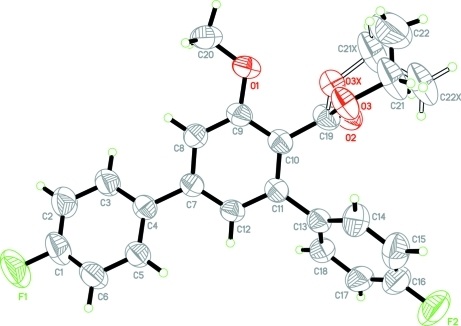
The molecular structure of the title compound with 50% probability displacement ellipsoids.

**Fig. 2.**
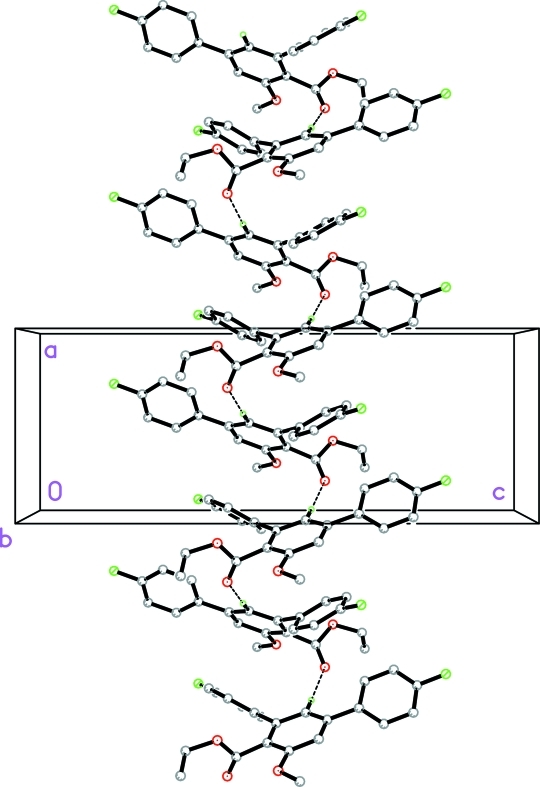
The crystal packing of the title compound viewed along the b axis. Only the major components of disorder are shown. Intermolecular hydrogen bonds are shown as dashed lines. Hydrogen atoms not involved in hydrogen bonding are omitted for clarity.

## supplementary crystallographic information

### Comment

In continuation of our work on the synthesis of terphenyl esters (Fun *et al.*, 2011; Samshuddin *et al.*, 2011), the title compound was prepared and its crystal structure is reported. The starting material of the title compound was prepared from 4,4'-difluoro chalcone by several steps.

The molecular structure of the title compound is shown in Fig. 1. The mean planes of the two fluoro-substituted phenyl rings (C1–C6 and C13–C18) make dihedral angles of 25.89 (15) and 55.00 (12)°, respectively, with the mean plane of the central benzene ring (C7–C12) in the terphenyl moiety. The ethoxy group (O3/C21/C22) is disordered over two positions with a site-occupancy ratio of 0.662 (7):0.338 (7). Bond lengths (Allen *et al.*, 1987) and angles are within normal ranges and are comparable to  those observed in a related structure (Fun *et al.*, 2011).

In the crystal structure (Fig. 2), the molecules are interconnected by C12—H12A···O2 hydrogen bonds (Table 1) into chains along the *a* axis. The crystal structure is further stabilized by C—H···π interactions (Table 1). π—π interactions are also observed with *Cg*_2_···*Cg*_3_ distance = 3.8605 (15) Å (symmetry code: 1/2+x,1/2-y,-z; *Cg*_2_ and *Cg*_3_ are the centroids of the C7–C12 and C13—C18 rings, respectively).

### Experimental

The title compound was prepared by the aromatization of a cyclohexenone derivative, ethyl 4,6-bis(4-fluorophenyl)-2-oxocyclohex-3-ene-1-carboxylate, using iodine and methanol at reflux condition (Kotnis, 1990). Single crystals of the product were grown from methanol by slow evaporation method (m.p. 383 K).

### Refinement

All H atoms were positioned geometrically [C—H = 0.93, 0.96 or 0.97 Å] and refined using a riding model with *U*_iso_(H) = 1.2 or 1.5*U*_eq_(C). A rotating group model was applied to the methyl group. The ethoxy unit was disordered over two positions with a site-occupancy ratio of 0.662 (7):0.338 (7). The same *U^ij^* parameters were used for atoms pairs C21/C21X and C22/C22X. The C13–C18 benzene ring was refined using a rigid regular hexagon model with a bond length of 1.39 Å. 2085 Friedel pairs were merged in the last refinement cycles.

### Crystal data

**Table:**

C_22_H_18_F_2_O_3_	*F*(000) = 768
*M**_r_* = 368.36	*D*_x_ = 1.319 Mg m^−^^3^
Orthorhombic, *P*2_1_2_1_2_1_	Mo *K*α radiation, λ = 0.71073 Å
Hall symbol: P 2ac 2ab	Cell parameters from 3149 reflections
*a* = 8.5197 (11) Å	θ = 2.3–22.9°
*b* = 9.5225 (12) Å	µ = 0.10 mm^−^^1^
*c* = 22.871 (3) Å	*T* = 296 K
*V* = 1855.5 (4) Å^3^	Block, colourless
*Z* = 4	0.43 × 0.21 × 0.15 mm

### Data collection

**Table:**

Bruker APEX DUO CCD area-detector diffractometer	3036 independent reflections
Radiation source: fine-focus sealed tube	2131 reflections with *I* > 2σ(*I*)
graphite	*R*_int_ = 0.026
φ and ω scans	θ_max_ = 30.0°, θ_min_ = 2.3°
Absorption correction: multi-scan (*SADABS*; Bruker, 2009)	*h* = −11→11
*T*_min_ = 0.958, *T*_max_ = 0.986	*k* = −13→9
11701 measured reflections	*l* = −26→32

### Refinement

**Table:**

Refinement on *F*^2^	Primary atom site location: structure-invariant direct methods
Least-squares matrix: full	Secondary atom site location: difference Fourier map
*R*[*F*^2^ > 2σ(*F*^2^)] = 0.057	Hydrogen site location: inferred from neighbouring sites
*wR*(*F*^2^) = 0.171	H-atom parameters constrained
*S* = 1.10	*w* = 1/[σ^2^(*F*_o_^2^) + (0.0841*P*)^2^ + 0.2095*P*] where *P* = (*F*_o_^2^ + 2*F*_c_^2^)/3
3036 reflections	(Δ/σ)_max_ = 0.011
251 parameters	Δρ_max_ = 0.20 e Å^−^^3^
0 restraints	Δρ_min_ = −0.23 e Å^−^^3^

### Special details

**Table:**

Geometry. All e.s.d.'s (except the e.s.d. in the dihedral angle between two l.s. planes) are estimated using the full covariance matrix. The cell e.s.d.'s are taken into account individually in the estimation of e.s.d.'s in distances, angles and torsion angles; correlations between e.s.d.'s in cell parameters are only used when they are defined by crystal symmetry. An approximate (isotropic) treatment of cell e.s.d.'s is used for estimating e.s.d.'s involving l.s. planes.
Refinement. Refinement of *F*^2^ against ALL reflections. The weighted *R*-factor *wR* and goodness of fit *S* are based on *F*^2^, conventional *R*-factors *R* are based on *F*, with *F* set to zero for negative *F*^2^. The threshold expression of *F*^2^ > σ(*F*^2^) is used only for calculating *R*-factors(gt) *etc*. and is not relevant to the choice of reflections for refinement. *R*-factors based on *F*^2^ are statistically about twice as large as those based on *F*, and *R*- factors based on ALL data will be even larger.

### Fractional atomic coordinates and isotropic or equivalent isotropic displacement parameters (Å^2^)

**Table:**

	*x*	*y*	*z*	*U*_iso_*/*U*_eq_	Occ. (<1)
F1	−0.2161 (4)	0.4152 (4)	−0.32486 (9)	0.1192 (10)	
F2	−0.0906 (3)	−0.0580 (3)	0.15941 (11)	0.1145 (10)	
O1	0.2205 (3)	0.7197 (2)	−0.00201 (11)	0.0713 (6)	
O2	0.3043 (3)	0.4532 (3)	0.09557 (11)	0.0776 (7)	
C1	−0.1600 (4)	0.4267 (5)	−0.26928 (13)	0.0778 (11)	
C2	−0.0345 (5)	0.5148 (5)	−0.25967 (14)	0.0806 (11)	
H2A	0.0111	0.5650	−0.2901	0.097*	
C3	0.0212 (4)	0.5260 (4)	−0.20305 (13)	0.0687 (9)	
H3A	0.1063	0.5843	−0.1954	0.082*	
C4	−0.0473 (3)	0.4518 (3)	−0.15723 (11)	0.0503 (6)	
C5	−0.1719 (3)	0.3640 (3)	−0.16957 (13)	0.0569 (7)	
H5A	−0.2183	0.3127	−0.1396	0.068*	
C6	−0.2285 (4)	0.3514 (4)	−0.22611 (14)	0.0708 (9)	
H6A	−0.3124	0.2920	−0.2343	0.085*	
C7	0.0110 (3)	0.4665 (3)	−0.09605 (11)	0.0477 (6)	
C8	0.0859 (3)	0.5911 (3)	−0.07870 (12)	0.0524 (6)	
H8A	0.0974	0.6644	−0.1052	0.063*	
C9	0.1428 (3)	0.6052 (3)	−0.02228 (13)	0.0528 (6)	
C10	0.1231 (3)	0.4972 (3)	0.01847 (12)	0.0502 (6)	
C11	0.0447 (3)	0.3755 (3)	0.00194 (12)	0.0485 (6)	
C12	−0.0081 (3)	0.3612 (3)	−0.05553 (12)	0.0493 (6)	
H12A	−0.0574	0.2783	−0.0667	0.059*	
C13	0.0119 (3)	0.26039 (16)	0.04462 (8)	0.0530 (6)	
C14	−0.0684 (3)	0.28881 (19)	0.09618 (9)	0.0701 (9)	
H14A	−0.0987	0.3803	0.1049	0.084*	
C15	−0.1035 (3)	0.1805 (3)	0.13478 (8)	0.0852 (11)	
H15A	−0.1572	0.1995	0.1693	0.102*	
C16	−0.0582 (3)	0.0438 (2)	0.12181 (10)	0.0783 (10)	
C17	0.0221 (3)	0.01533 (15)	0.07024 (10)	0.0765 (10)	
H17A	0.0524	−0.0762	0.0616	0.092*	
C18	0.0571 (3)	0.12364 (19)	0.03165 (8)	0.0649 (8)	
H18A	0.1109	0.1046	−0.0029	0.078*	
C19	0.1921 (4)	0.5144 (3)	0.07789 (14)	0.0601 (7)	
C20	0.2508 (5)	0.8306 (3)	−0.04159 (18)	0.0758 (10)	
H20A	0.3045	0.9051	−0.0216	0.114*	
H20B	0.1533	0.8653	−0.0570	0.114*	
H20C	0.3150	0.7968	−0.0731	0.114*	
O3	0.0943 (6)	0.5935 (6)	0.1127 (2)	0.0681 (13)	0.662 (7)
C21	0.1430 (9)	0.6182 (10)	0.1721 (3)	0.101 (2)	0.662 (7)
H21A	0.0502	0.6354	0.1956	0.122*	0.662 (7)
H21B	0.1920	0.5333	0.1868	0.122*	0.662 (7)
C22	0.2433 (17)	0.7258 (11)	0.1799 (5)	0.162 (5)	0.662 (7)
H22A	0.2898	0.7191	0.2181	0.243*	0.662 (7)
H22B	0.1875	0.8130	0.1766	0.243*	0.662 (7)
H22C	0.3241	0.7219	0.1507	0.243*	0.662 (7)
O3X	0.1537 (10)	0.6375 (9)	0.1002 (4)	0.0542 (19)	0.338 (7)
C21X	0.231 (2)	0.674 (2)	0.1527 (5)	0.101 (2)	0.338 (7)
H21C	0.2259	0.7743	0.1595	0.122*	0.338 (7)
H21D	0.3405	0.6455	0.1515	0.122*	0.338 (7)
C22X	0.1341 (17)	0.586 (2)	0.2049 (6)	0.101 (2)	0.338 (7)
H22D	0.1894	0.5948	0.2413	0.152*	0.338 (7)
H22E	0.1269	0.4888	0.1943	0.152*	0.338 (7)
H22F	0.0305	0.6246	0.2090	0.152*	0.338 (7)

### Atomic displacement parameters (Å^2^)

**Table:**

	*U*^11^	*U*^22^	*U*^33^	*U*^12^	*U*^13^	*U*^23^
F1	0.1238 (19)	0.184 (3)	0.0495 (10)	−0.031 (2)	−0.0206 (13)	−0.0071 (15)
F2	0.1106 (19)	0.1178 (18)	0.115 (2)	−0.0261 (17)	−0.0049 (16)	0.0635 (17)
O1	0.0911 (15)	0.0529 (12)	0.0699 (13)	−0.0167 (12)	−0.0212 (13)	−0.0061 (11)
O2	0.0827 (15)	0.0703 (13)	0.0798 (15)	0.0215 (13)	−0.0310 (13)	−0.0108 (12)
C1	0.074 (2)	0.114 (3)	0.0449 (15)	−0.001 (2)	−0.0088 (15)	−0.0080 (18)
C2	0.084 (2)	0.107 (3)	0.0504 (17)	−0.017 (2)	0.0000 (16)	0.0070 (18)
C3	0.0666 (18)	0.089 (2)	0.0507 (15)	−0.0189 (18)	−0.0031 (14)	0.0025 (16)
C4	0.0518 (13)	0.0517 (14)	0.0472 (13)	0.0015 (12)	−0.0013 (11)	−0.0051 (12)
C5	0.0565 (14)	0.0635 (17)	0.0508 (15)	−0.0046 (14)	−0.0029 (12)	−0.0042 (13)
C6	0.0681 (18)	0.085 (2)	0.0596 (18)	−0.0133 (18)	−0.0115 (15)	−0.0120 (17)
C7	0.0479 (12)	0.0482 (13)	0.0469 (13)	−0.0001 (11)	−0.0008 (11)	−0.0041 (11)
C8	0.0566 (14)	0.0483 (14)	0.0522 (14)	0.0011 (12)	−0.0040 (12)	0.0003 (12)
C9	0.0550 (13)	0.0480 (15)	0.0553 (15)	−0.0007 (12)	−0.0057 (12)	−0.0081 (12)
C10	0.0521 (13)	0.0463 (13)	0.0522 (14)	0.0074 (11)	−0.0063 (11)	−0.0060 (11)
C11	0.0507 (12)	0.0452 (13)	0.0495 (13)	0.0053 (11)	−0.0055 (11)	−0.0033 (11)
C12	0.0523 (13)	0.0455 (13)	0.0501 (13)	−0.0040 (12)	−0.0024 (11)	−0.0061 (11)
C13	0.0533 (13)	0.0534 (14)	0.0524 (14)	−0.0017 (12)	−0.0082 (12)	0.0002 (12)
C14	0.0736 (19)	0.073 (2)	0.0636 (18)	0.0121 (18)	0.0029 (16)	0.0092 (16)
C15	0.071 (2)	0.119 (3)	0.066 (2)	0.004 (2)	0.0070 (18)	0.017 (2)
C16	0.0675 (18)	0.083 (2)	0.085 (2)	−0.0157 (19)	−0.0109 (18)	0.030 (2)
C17	0.089 (2)	0.0574 (18)	0.083 (2)	−0.0118 (18)	−0.013 (2)	0.0115 (17)
C18	0.0804 (19)	0.0503 (15)	0.0639 (18)	−0.0037 (15)	−0.0085 (16)	0.0022 (14)
C19	0.0694 (17)	0.0488 (15)	0.0621 (17)	0.0097 (14)	−0.0164 (14)	−0.0082 (13)
C20	0.084 (2)	0.0539 (17)	0.090 (2)	−0.0160 (17)	−0.009 (2)	−0.0042 (17)
O3	0.067 (3)	0.088 (3)	0.049 (2)	0.010 (2)	−0.006 (2)	−0.020 (2)
C21	0.093 (3)	0.159 (7)	0.053 (3)	0.015 (4)	−0.010 (3)	−0.033 (4)
C22	0.229 (13)	0.114 (7)	0.142 (9)	−0.014 (8)	−0.090 (9)	−0.010 (6)
O3X	0.054 (4)	0.059 (4)	0.049 (4)	0.004 (3)	−0.003 (3)	−0.004 (3)
C21X	0.093 (3)	0.159 (7)	0.053 (3)	0.015 (4)	−0.010 (3)	−0.033 (4)
C22X	0.093 (3)	0.159 (7)	0.053 (3)	0.015 (4)	−0.010 (3)	−0.033 (4)

### Geometric parameters (Å, °)

**Table:**

F1—C1	1.362 (4)	C14—C15	1.3900
F2—C16	1.325 (3)	C14—H14A	0.9300
O1—C9	1.357 (3)	C15—C16	1.3900
O1—C20	1.415 (4)	C15—H15A	0.9300
O2—C19	1.191 (4)	C16—C17	1.3900
C1—C6	1.353 (5)	C17—C18	1.3900
C1—C2	1.376 (5)	C17—H17A	0.9300
C2—C3	1.383 (5)	C18—H18A	0.9300
C2—H2A	0.9300	C19—O3X	1.320 (9)
C3—C4	1.392 (4)	C19—O3	1.377 (5)
C3—H3A	0.9300	C20—H20A	0.9600
C4—C5	1.381 (4)	C20—H20B	0.9600
C4—C7	1.491 (4)	C20—H20C	0.9600
C5—C6	1.385 (4)	O3—C21	1.440 (8)
C5—H5A	0.9300	C21—C22	1.346 (14)
C6—H6A	0.9300	C21—H21A	0.9700
C7—C12	1.375 (4)	C21—H21B	0.9700
C7—C8	1.404 (4)	C22—H22A	0.9600
C8—C9	1.385 (4)	C22—H22B	0.9600
C8—H8A	0.9300	C22—H22C	0.9600
C9—C10	1.398 (4)	O3X—C21X	1.414 (16)
C10—C11	1.390 (4)	C21X—C22X	1.68 (3)
C10—C19	1.490 (4)	C21X—H21C	0.9700
C11—C12	1.396 (4)	C21X—H21D	0.9700
C11—C13	1.494 (3)	C22X—H22D	0.9600
C12—H12A	0.9300	C22X—H22E	0.9600
C13—C14	1.3900	C22X—H22F	0.9600
C13—C18	1.3900		
			
C9—O1—C20	118.0 (2)	C16—C15—C14	120.0
C6—C1—F1	119.2 (3)	C16—C15—H15A	120.0
C6—C1—C2	122.8 (3)	C14—C15—H15A	120.0
F1—C1—C2	118.0 (3)	F2—C16—C17	120.7 (2)
C1—C2—C3	117.6 (3)	F2—C16—C15	119.3 (2)
C1—C2—H2A	121.2	C17—C16—C15	120.0
C3—C2—H2A	121.2	C16—C17—C18	120.0
C2—C3—C4	121.5 (3)	C16—C17—H17A	120.0
C2—C3—H3A	119.3	C18—C17—H17A	120.0
C4—C3—H3A	119.3	C17—C18—C13	120.0
C5—C4—C3	118.4 (3)	C17—C18—H18A	120.0
C5—C4—C7	120.3 (3)	C13—C18—H18A	120.0
C3—C4—C7	121.3 (3)	O2—C19—O3X	120.2 (5)
C6—C5—C4	120.7 (3)	O2—C19—O3	123.9 (3)
C6—C5—H5A	119.6	O2—C19—C10	125.0 (3)
C4—C5—H5A	119.6	O3X—C19—C10	110.6 (4)
C1—C6—C5	119.0 (3)	O3—C19—C10	110.4 (3)
C1—C6—H6A	120.5	O1—C20—H20A	109.5
C5—C6—H6A	120.5	O1—C20—H20B	109.5
C12—C7—C8	118.6 (2)	H20A—C20—H20B	109.5
C12—C7—C4	121.6 (2)	O1—C20—H20C	109.5
C8—C7—C4	119.7 (2)	H20A—C20—H20C	109.5
C9—C8—C7	120.3 (3)	H20B—C20—H20C	109.5
C9—C8—H8A	119.9	C19—O3—C21	117.4 (5)
C7—C8—H8A	119.9	C22—C21—O3	115.7 (10)
O1—C9—C8	124.6 (3)	C22—C21—H21A	108.4
O1—C9—C10	114.9 (2)	O3—C21—H21A	108.4
C8—C9—C10	120.5 (3)	C22—C21—H21B	108.4
C11—C10—C9	119.3 (2)	O3—C21—H21B	108.4
C11—C10—C19	122.0 (2)	H21A—C21—H21B	107.4
C9—C10—C19	118.7 (2)	C19—O3X—C21X	115.5 (10)
C10—C11—C12	119.5 (2)	O3X—C21X—C22X	104.7 (15)
C10—C11—C13	121.6 (2)	O3X—C21X—H21C	110.8
C12—C11—C13	118.9 (2)	C22X—C21X—H21C	110.8
C7—C12—C11	121.7 (2)	O3X—C21X—H21D	110.8
C7—C12—H12A	119.2	C22X—C21X—H21D	110.8
C11—C12—H12A	119.2	H21C—C21X—H21D	108.9
C14—C13—C18	120.0	C21X—C22X—H22D	109.5
C14—C13—C11	120.24 (15)	C21X—C22X—H22E	109.5
C18—C13—C11	119.72 (15)	H22D—C22X—H22E	109.5
C13—C14—C15	120.0	C21X—C22X—H22F	109.5
C13—C14—H14A	120.0	H22D—C22X—H22F	109.5
C15—C14—H14A	120.0	H22E—C22X—H22F	109.5
			
C6—C1—C2—C3	−0.5 (6)	C10—C11—C12—C7	−2.1 (4)
F1—C1—C2—C3	179.9 (4)	C13—C11—C12—C7	176.5 (2)
C1—C2—C3—C4	−0.4 (6)	C10—C11—C13—C14	55.1 (3)
C2—C3—C4—C5	1.1 (5)	C12—C11—C13—C14	−123.4 (2)
C2—C3—C4—C7	−178.7 (3)	C10—C11—C13—C18	−127.1 (2)
C3—C4—C5—C6	−0.8 (5)	C12—C11—C13—C18	54.4 (3)
C7—C4—C5—C6	178.9 (3)	C18—C13—C14—C15	0.0
F1—C1—C6—C5	−179.6 (4)	C11—C13—C14—C15	177.8 (2)
C2—C1—C6—C5	0.7 (6)	C13—C14—C15—C16	0.0
C4—C5—C6—C1	−0.1 (6)	C14—C15—C16—F2	179.1 (2)
C5—C4—C7—C12	26.1 (4)	C14—C15—C16—C17	0.0
C3—C4—C7—C12	−154.1 (3)	F2—C16—C17—C18	−179.1 (2)
C5—C4—C7—C8	−153.1 (3)	C15—C16—C17—C18	0.0
C3—C4—C7—C8	26.6 (4)	C16—C17—C18—C13	0.0
C12—C7—C8—C9	1.7 (4)	C14—C13—C18—C17	0.0
C4—C7—C8—C9	−179.0 (2)	C11—C13—C18—C17	−177.8 (2)
C20—O1—C9—C8	−2.3 (5)	C11—C10—C19—O2	70.4 (4)
C20—O1—C9—C10	177.3 (3)	C9—C10—C19—O2	−107.4 (4)
C7—C8—C9—O1	178.1 (3)	C11—C10—C19—O3X	−132.9 (5)
C7—C8—C9—C10	−1.5 (4)	C9—C10—C19—O3X	49.3 (6)
O1—C9—C10—C11	179.9 (2)	C11—C10—C19—O3	−100.0 (4)
C8—C9—C10—C11	−0.4 (4)	C9—C10—C19—O3	82.2 (4)
O1—C9—C10—C19	−2.2 (4)	O2—C19—O3—C21	8.6 (9)
C8—C9—C10—C19	177.4 (3)	O3X—C19—O3—C21	−84.6 (11)
C9—C10—C11—C12	2.2 (4)	C10—C19—O3—C21	179.1 (6)
C19—C10—C11—C12	−175.6 (3)	C19—O3—C21—C22	83.5 (10)
C9—C10—C11—C13	−176.3 (2)	O2—C19—O3X—C21X	−13.1 (13)
C19—C10—C11—C13	5.9 (4)	O3—C19—O3X—C21X	93.4 (14)
C8—C7—C12—C11	0.1 (4)	C10—C19—O3X—C21X	−171.1 (10)
C4—C7—C12—C11	−179.2 (2)	C19—O3X—C21X—C22X	−79.9 (12)

### Hydrogen-bond geometry (Å, °)

**Table:**

Cg2 and Cg3 are the centroids of the C7–C12 and C13–C18 rings, respectively.

**Table:**

*D*—H···*A*	*D*—H	H···*A*	*D*···*A*	*D*—H···*A*
C12—H12A···O2^i^	0.93	2.59	3.515 (3)	179.
C5—H5A···Cg3^ii^	0.93	2.92	3.589 (3)	130
C20—H20A···Cg2^iii^	0.96	2.83	3.710 (4)	152

Symmetry codes: (i) *x*−1/2, −*y*+1/2, −*z*; (ii) −*x*−1, *y*+1/2, −*z*+1/2; (iii) −*x*, *y*+3/2, −*z*+1/2.
